# Breast Subtypes and Prognosis of Breast Cancer Patients With Initial Bone Metastasis: A Population-Based Study

**DOI:** 10.3389/fonc.2020.580112

**Published:** 2020-12-02

**Authors:** Deyue Liu, Jiayi Wu, Caijin Lin, Lisa Andriani, Shuning Ding, Kunwei Shen, Li Zhu

**Affiliations:** Department of General Surgery, Comprehensive Breast Health Center, Ruijin Hospital, Shanghai Jiaotong University School of Medicine, Shanghai, China

**Keywords:** *de novo* stage IV, breast cancer, bone metastase, nomogram, prediction

## Abstract

**Background:**

Metastatic breast cancer (MBC) is a highly heterogeneous disease and bone is one of the most common metastatic sites. This retrospective study was conducted to investigate the clinical features, prognostic factors and benefits of surgery of breast cancer patients with initial bone metastases.

**Methods:**

From 2010 to 2015, 6,860 breast cancer patients diagnosed with initial bone metastasis were analyzed from Surveillance, Epidemiology, and End Results (SEER) database. Univariate and Multivariable analysis were used to identify prognostic factors. A nomogram was performed based on the factors selected from cox regression result. Survival curves were plotted according to different subtypes, metastatic burdens and risk groups differentiated by nomogram.

**Results:**

Hormone receptor (HR) positive/human epidermal growth factor receptor 2 (HER2) positive patients showed the best outcome compared to other subtypes. Patients of younger age (<60 years old), white race, lower grade, lower T stage (<=T2), not combining visceral metastasis tended to have better outcome. About 37% (2,249) patients received surgery of primary tumor. Patients of all subtypes could benefit from surgery. Patients of bone-only metastases (BOM), bone and liver metastases, bone and lung metastases also showed superior survival time if surgery was performed. However, patients of bone and brain metastasis could not benefit from surgery (p = 0.05). The C-index of nomogram was 0.66. Cutoff values of nomogram point were identified as 87 and 157 points, which divided all patients into low-, intermediate- and high-risk groups. Patients of all groups showed better overall survival when receiving surgery.

**Conclusion:**

Our study has provided population-based prognostic analysis in patients with initial bone metastatic breast cancer and constructed a predicting nomogram with good accuracy. The finding of potential benefit of surgery to overall survival will cast some lights on the treatment tactics of this group of patients.

## Introduction

Breast cancer is the most commonly diagnosed malignant tumor and the leading cause of cancer death among females worldwide, accounting for 24.2% of all new cases and 15.0% of cases of death (1). Approximately 5–8% of breast cancer patients demonstrate distant metastasis at first diagnosis (2). *De novo* stage IV breast cancer is usually considered an incurable disease. The overall 5-year breast cancer specific survival (BCSS) of *de novo* stage IV breast cancer patients is about 27%. However, with the advance of systemic therapy and local treatment, the prognosis has been largely improved (3, 4).

Metastatic breast cancer (MBC) is a highly heterogeneous disease with a wide range of clinical manifestation from solitary to multiple visceral involvements. Metastatic pattern is highly correlated to breast cancer subtype. Patients with hormone receptor positive and human epidermal growth factor receptor 2 negative (HR^+^/HER2^−^) disease were reported to have more bone metastasis, patients with HR^−^/HER2^+^ tumors had more liver metastasis, whereas brain and lung metastasis were more likely to occur in HR^−^/HER2^−^ patients (5, 6). Bone metastases, whether oligometastatic or combined with metastasis to other sites, were most commonly diagnosed, representing around 70% in MBC patients (7, 8). Patients with bone metastasis exhibited preferred prognosis compared with visceral metastasis due to different metastatic pattern of different subtypes (6, 9). Even though, different subtypes and metastatic patterns presented divergent outcomes. Previous analysis showed that patients of bone-only metastasis and HR^+^/HER2^−^ subtype better overall survival (OS) (10, 11).

Therapeutic goals in MBC are usually maintenance of quality of life and palliation of symptoms. Generally, systemic therapy is the primary choice including chemotherapy, targeted therapy, endocrine therapy and immune therapy. It is still controversial about the role of surgery in metastatic patients. Therefore, surgery for MBC patients is a choice but not a preference for now with the existing evidence. Subgroup analyses of several retrospective trials have suggested a prolonged survival time for bone metastatic patients, while others turned out just the opposite.

The purpose of our study was to investigate the prognostic factors of *de novo* stage IV breast cancer patients with bone metastasis and if surgery of the primary site could benefit them.

## Patients and Methods

### Data Collection

The data were extracted from the SEER database. Patients diagnosed of breast cancer with *de novo* bone metastasis from 2010 to 2015 with active follow-up, valid survival time, known subtype information, known American Joint Committee on Cancer (AJCC) system stage, *de novo* bone involvement, known visceral metastatic status, known surgery of the primary site, known cause of death were included. Patients with other malignant comorbidities were excluded to eliminate the effect of other malignancy to OS. Occult breast cancer patients (T0), undefined T and N stage patients were excluded from the analysis. Patients diagnosed only in autopsy and death certification were also excluded. At last, 6,860 patients were included in the analysis.

Before initiating this study, we submitted a data-use agreement to the SEER program and were officially granted access to the database. The variables extracted were age at diagnosis (<60 and ≥60 years old), race (white, black, other >and unknown), gender (female and male), year of diagnosis (2010, 2011, 2012, 2013, 2014, 2015), breast subtypes (HR^+^/HER2^−^, HR^+^/HER2^+^, HR^−^/HER2^+^ and HR^−^/HER2^−^ subtypes), grade (I, II, III, IV, unknown), derived American Joint Committee on Cancer (AJCC) T stage (T1, T2, T3, T4), derived AJCC N stage (N0, N1, N2, N3), marital status at diagnosis (married, unmarried and unknown), insurance status (insured, uninsured and unknown), brain metastasis status at diagnosis (yes or no), liver metastasis status at diagnosis (yes or no), lung metastasis status at diagnosis (yes or no), SEER cause-specific death classification (alive or dead of other cause and dead attributable to this cancer), vital status (alive and dead), survival time and surgery information of primary site.

### Statistical Analysis

The frequency and proportion of the baseline characteristics in the study cohort were by described by chi-square test. OS and BCSS were both calculated to evaluate prognosis. Univariate analysis was performed with variables including age, sex, race, grade, subtype, T stage, N stage, marital status, insurance status, visceral metastases and surgery or not. The statistically meaningful (p <0.05) variables were taken into the multivariable Cox analysis to determine the independent prognostic factors of patients with bone involvement. Kaplan–Meier survival curves were plotted to estimate the OS and BCSS. Log-rank test was applied in comparing survival. A nomogram model based on the statistically significant factors in multivariate analysis was plotted to predict a patient of specific characteristic. A concordance index (c-index) was calculated to evaluate the performance of the nomogram. Calibration curves were plotted to evaluate the consistency between predicted and actual overall survival at 3 and 5 years, respectively. The cutoff values were generated by X-tile software (3.6.1; https://medicine.yale.edu/lab/rimm/research/software). All statistical analyses were carried out with R software (version 3.6.1; http://www.R-project.org). A two-tailed p <0.05 was considered statistical significant.

## Results

### Baseline Characteristics

The demographic and clinical characteristic of *de novo* metastatic breast cancer patients with bone involvement were shown in **Table 1**. Among the total cohort, 67.06% (4,600/6,860), 17.38% (1,192/6,860), 6.52% (447/6,860), 9.05% (621/6,860) of the patients had HR^+^/HER2^−^, HR^+^/HER2^+^, HR^−^/HER2^+^, HR^−^/HER2^−^ tumors respectively. Patients with HR^+^/HER2^−^ tumors tended to be older and lymph node-negative. Patients with HR^+^/HER2^+^ and HR^−^/HER2^+^ tumors had a higher grade and T stage. Patients with HR^−^/HER2^+^ tumors had increased incidences of brain metastases (HR^+^/HER2^−^ vs HR^+^/HER2^+^ vs HR^−^/HER2^+^ vs HR^−^/HER2^−^: 4.96 vs 7.97% vs 11.63 vs 10.47%, p <0.001), liver metastases (HR^+^/HER2^−^ vs HR^+^/HER2^+^ vs HR^−^/HER2^+^ vs HR^−^/HER2^−^: 15.93 vs 32.21% vs 45.19 vs 29.79%, p <0.001) and lung metastases (HR^+^/HER2^−^ vs HR^+^/HER2^+^ vs HR^−^/HER2^+^ vs HR^−^/HER2^−^: 22.96 vs 26.76% vs 31.32 vs 29.47%, p <0.001).

**Table 1:** Demographic and clinical characteristics of *de novo* IV patients with bone metastasis grouped by subtypes.

	All subtypes n (%) N = 6860	HR^+^/HER2^−^ n (%) N = 4600	HR^+^/HER2^+^ n (%) N = 1192	HR^−^/HER2^+^ n (%) N = 447	HR^−^/HER2^−^ n (%) N = 621	p
**Age, y**						
<60	3,454 (50.35)	2,136 (46.43)	714 (59.90)	283 (63.31)	321 (51.69)	<0.001
>=60	3,406 (49.65)	2,464 (53.57)	478 (40.10)	164 (36.69)	300 (48.31)	
**Sex**						
Female	6,768 (98.66)	4,534 (98.57)	1,173 (98.41)	445 (99.55)	616 (99.19)	0.177
Male	92 (1.34)	66 (1.43)	19 (1.59)	2 (0.45)	5 (0.81)	
**Race**						
White	5,178 (75.61)	3,550 (77.17)	888 (74.50)	321 (71.81)	428 (68.92)	<0.001
Black	1,127 (16.43)	681 (14.80)	207 (17.37)	81 (18.12)	158 (25.44)	
Other**^(1)^**	532 (7.76)	358 (7.78)	96 (8.05)	44 (9.84)	34 (5.48)	
Unknown	14 (0.20)	11 (0.24)	1 (0.08)	1 (0.22)	1 (0.16)	
**Histologic grade**						
I	508 (7.41)	472 (10.26)	24 (2.01)	3 (0.67)	9 (1.45)	<0.001
II	2,761 (40.25)	2,121 (46.17)	430 (36.07)	100 (22.37)	107 (17.23)	
III	2,603 (37.94)	1,320 (28.70)	576 (48.32)	276 (61.74)	431 (69.40)	
IV**^(2)^**	24 (0.35)	13 (0.28)	1 (0.08)	2 (0.45)	8 (1.29)	
Unknown	964 (14.05)	671 (14.59)	161 (13.51)	66 (14.77)	66 (10.63)	
**AJCC T stage**						
1	844 (12.30)	598 (13)	138 (11.58)	44 (9.84)	64 (10.31)	<0.001
2	2,345 (34.18)	1,647 (35.80)	397 (33.31)	118 (26.40)	183 (29.47)	
3	1,276 (18.60)	868 (18.87)	206 (17.28)	85 (19.02)	117 (18.84)	
4	2,395 (34.91)	1,487 (32.33)	451 (37.84)	200 (44.74)	257 (41.38)	
**AJCC N stage**						
0	1,486 (21.66)	1,067 (23.20)	233 (19.55)	64 (14.32)	122 (19.65)	<0.001
1	3,356 (48.92)	2,220 (48.26)	601 (50.42)	233 (52.13)	302 (48.63)	
2	929 (13.54)	642 (13.96)	151 (12.67)	61 (13.65)	75 (12.08)	
3	1,089 (15.87)	671 (14.59)	207 (17.37)	89 (19.91)	122 (19.65)	
**Marital status**						
Married	3135 (45.70)	2096(45.57)	548(45.97)	219(48.99)	272(43.80)	0.268
Unmarried**^(3)^**	3386(49.36)	2276(49.48)	578(48.49)	205(45.86)	327(52.66)	
Unknown	339(4.94)	228(4.96)	66(5.54)	23(5.15)	22(3.54)	
**Insurance status**						
Insured**^(4)^**	6,488 (94.58)	4,361 (94.80)	1113 (93.37)	425 (95.08)	589 (94.85)	0.625
Uninsured	269 (3.92)	171 (3.72)	58 (4.87)	16 (3.58)	24 (3.86)	
Unknown	103 (1.50)	68 (1.48)	21 (1.76)	6 (1.34)	8 (1.29)	
**Brain involvement**						
No	6,420 (93.59)	4,372 (95.04)	1,097 (92.03)	395 (88.37)	556 (89.53)	<0.001
Yes	440 (6.41)	228 (4.96)	95 (7.97)	52 (11.63)	65 (10.47)	
**Liver involvement**						
No	5,356 (78.08)	3,867 (84.07)	808 (67.79)	245 (54.81)	436 (70.21)	<0.001
Yes	1,504 (21.92)	733 (15.93)	384 (32.21)	202 (45.19)	185 (29.79)	
**Lung involvement**						
No	5,162 (75.25)	3,544 (77.04)	873 (73.24)	307 (68.68)	438 (70.53)	<0.001
Yes	1,698 (24.75)	1,056 (22.96)	319 (26.76)	140 (31.32)	183 (29.47)	
**Surgery**						
No	4,611 (67.22)	3,122 (67.87)	810 (67.95)	292 (65.32)	387 (62.32)	0.034
Yes	2,249 (32.78)	1,478 (32.13)	382 (32.05)	155 (34.68)	234 (37.68)	

^(1)^including American Indian/AK Native, Asian/Pacific Islander; ^(2)^including undifferentiated; anaplastic; Grade IV; ^(3)^including any Medicaid, insured or insured non-specifics; ^(4)^including divorced, single (never married), unmarried or domestic partner, widowed and separated.

HR, hormone receptor; HER2, human epidermal growth factor 2; AJCC, American Joint Committee on Cancer.

In HR^+^/HER2^−^ subgroup, lung was the most susceptible organ in initial bone involved patients, while in HR^+^/HER2^+^, HR^−^/HER2^+^ and HR^−^/HER2^−^ subgroups, concurrent liver involvement was the most common.

### Univariable and Multivariable Analysis

In univariate analysis, we found that patients of older age, black race, higher grade tumors, HR^−^/HER2^−^ subtype, high T stage (T >2), unmarried status, uninsured status, visceral involvement (brain, liver or lung), no primary tumor surgery displayed worse OS (**Table 2**).

**Table 2:** Univariate and multivariate cox progression of OS and BCSS of breast cancer patients with initial bone metastasis.

	Overall survival				Breast cancer-specific survival			
	Univariate analysis		Multivariate analysis		Univariate analysis		Multivariate analysis	
	HR (95% CI)	P	HR (95% CI)	P	HR (95% CI)	P	HR (95% CI)	P
**Age**								
<60	Reference				Reference			
≥60	1.40(1.31–1.49)	<0.001	1.43(1.33–1.52)	<0.001	1.32(1.24–1.41)	<0.001	1.36(1.27–1.45)	<0.001
**Sex**								
Female	Reference				Reference			
Male	1.15(0.88–1.51)	0.302	/	/	1.11(0.83–1.48)	0.479	/	/
**Race**								
White	Reference				Reference			
Black	1.42(1.31–1.54)	<0.001	1.27(1.16–1.38)	<0.001	1.41(1.29–1.53)	<0.001	1.24(1.13–1.35)	<0.001
Other	0.92(1.81–1.04)	0.172	0.93(0.82–1.05)	0.244	0.95(0.83–1.08)	0.432	0.95(0.84–1.09)	0.465
Unknown	0.15(0.02–1.08)	0.059	0.11(0.01–0.75)	0.025	0.16(0.02–1.17)	0.071	0.12(0.02–0.83)	0.031
**Grade**								
I	Reference				Reference			
II	1.28(1.11–1.48)	0.001	1.30(1.12–1.50)	<0.001	1.33(1.14–1.55)	<0.001	1.34(1.14–1.56)	<0.001
III	1.81(1.57–2.09)	<0.001	1.75(1.51–2.03)	<0.001	1.95(1.68–2.27)	<0.001	1.86(1.59–2.17)	<0.001
IV	4.04(2.62–6.21)	<0.001	2.42(1.57–3.74)	<0.001	4.64(3.01–7.15)	<0.001	2.75(1.78–4.27)	<0.001
Unknown	1.72(1.47–2.01)	<0.001	1.42(1.21–1.66)	<0.001	1.81(1.53–2.14)	<0.001	1.49(1.25–1.76)	<0.001
**Subtype**								
HR+/HER2-	Reference				Reference			
HR+/HER2+	0.80(0.73–0.88)	<0.001	0.65(0.59–0.72)	<0.001	0.82(0.75–0.91)	<0.001	0.65(0.59–0.72)	<0.001
HR-/HER2+	0.98(0.86–1.13)	0.8	0.74(0.64–0.86)	<0.001	1.02(0.88–1.17)	0.832	0.74(0.64–0.85)	<0.001
HR-/HER2-	2.94(2.67–3.23)	<0.001	2.51(2.27–2.78)	<0.001	3.03(2.74–3.34)	<0.001	2.54(2.28–2.82)	<0.001
**T**								
1	Reference				Reference			
2	0.98(0.88–1.10)	0.721	1.04(0.93–1.16)	0.527	0.96(0.85–1.07)	0.458	1.01(0.90–1.13)	0.893
3	1.19(1.05–1.34)	0.005	1.19(1.05–1.34)	0.005	1.19(1.05–1.35)	0.006	1.19(1.05–1.35)	0.008
4	1.49(1.33–1.66)	<0.001	1.27(1.14–1.42)	<0.001	1.48(1.33–1.66)	<0.001	1.26(1.12–1.41)	<0.001
**N**								
0	Reference				Reference			
1	0.97(0.89–1.05)	0.464	/	/	0.99(0.91–1.08)	0.829	/	/
2	0.97(0.87–1.08)	0.607	/	/	0.98(0.87–1.10)	0.702	/	/
3	1.03(0.93–1.14)	0.618	/	/	1.06(0.95–1.18)	0.287	/	/
**Marital status**								
Married	Reference				Reference			
Unmarried	1.36(1.27–1.45)	<0.001	1.23(1.15–1.32)	<0.001	1.33(1.24–1.42)	<0.001	1.21(1.13–1.30)	<0.001
Unknown	1.11(0.95–1.30)	0.17	1.05(0.90–1.23)	0.546	1.14(0.97–1.33)	0.113	1.07(0.91–1.26)	0.396
**Insurance**								
Insured	Reference				Reference			
Uninsured	1.27(1.09–1.48)	0.002	1.20(1.03–1.40)	0.021	1.34(1.14–1.56)	<0.001	1.26(1.07–1.47)	0.004
Unknown	0.96(0.73–1.25)	0.744	0.89(0.67–1.17)	0.393	0.98(0.75–1.3)	0.902	0.90(0.68–1.20)	0.480
**Brain involvement**								
No	Reference				Reference			
Yes	2.31(2.07–2.59)	<0.001	1.83(1.63–2.05)	<0.001	2.37(2.12–2.66)	<0.001	1.85(1.64–2.08)	<0.001
**Liver involvement**								
No	Reference				Reference			
Yes	1.83(1.71–1.97)	<0.001	1.68(1.56–1.82)	<0.001	1.93(1.79–2.08)	<0.001	1.75(1.62–1.90)	<0.001
**Lung involvement**								
No	Reference				Reference			
Yes	1.60(1.5–1.72)	<0.001	1.22(1.14–1.32)	<0.001	1.62(1.50–1.74)	<0.001	1.22(1.13–1.32)	<0.001
**Surgery**								
No	Reference				Reference			
Yes	0.56(0.5–0.60)	<0.001	0.60(0.56–0.65)	<0.001	0.56(0.52–0.60)	<0.001	0.60(0.56–0.65)	<0.001

HR, hormone receptor; HER2, human epidermal growth factor 2; AJCC, American Joint Committee on Cancer; CI, confidence interval.

These statistically significant factors were included in the multivariate analysis. Patients older than 60 years old (HR = 1.43, 95% CI = 1.33–1.52, p <0.001), black race (HR = 1.27, 95% CI = 1.16–1.38, p <0.001), T3 stage (T2 vs T1: HR = 1.04, 95% CI = 0.93–1.16, p = 0.527; T3 vs T1: HR = 1.19, 95% CI = 1.05–1.34, p = 0.005) were significantly related to worse OS. Compared with HR^+^/HER2^−^ patients, HR^+^/HER2^+^ and HR^−^/HER2^+^ subtype showed improved OS (HR^+^/HER2^+^: HR = 0.65, 95% CI = 0.59–0.72, p <0.001; HR^−^/HER2^+^: HR = 0.74, 95% CI = 0.64–0.86, p <0.001), while HR^−^/HER2^−^ subtype demonstrated the worst outcome (HR = 2.51, 95% CI = 2.27–2.78, p <0.001). Social factors like marital status (HR = 1.23, 95% CI = 1.15–1.32, p <0.001) and insurance status (HR = 1.2, 95% CI = 1.03–1.4, p <0.001) were also associated with OS.

Among the 6,860 patients with bone metastatic lesions, 4096 cases (59.71%) demonstrated bone-only metastasis and 2,764 cases (40.29%) displayed concurrent visceral metastases. The outcome was much worse when combining visceral metastases (BOM vs bone and brain metastasis: median OS = 43 vs 17 months, HR = 1.83, 95% CI = 1.63–2.05, p <0.001; BOM vs bone and liver metastasis: median OS = 43 vs 27 months, HR = 1.68, 95% CI = 1.56–1.82, p <0.001; BOM vs bone and lung metastasis is: median OS = 43 vs 31 months, HR = 1.22, 95% CI = 1.14–1.32, p <0.001). In terms of BCSS, univariate and multivariate results identified the same prognostic factors as OS (**Table 2**).

### Development and Validation of a 3-Year and 5-Year OS Predicting Nomogram

**Figure 1:**
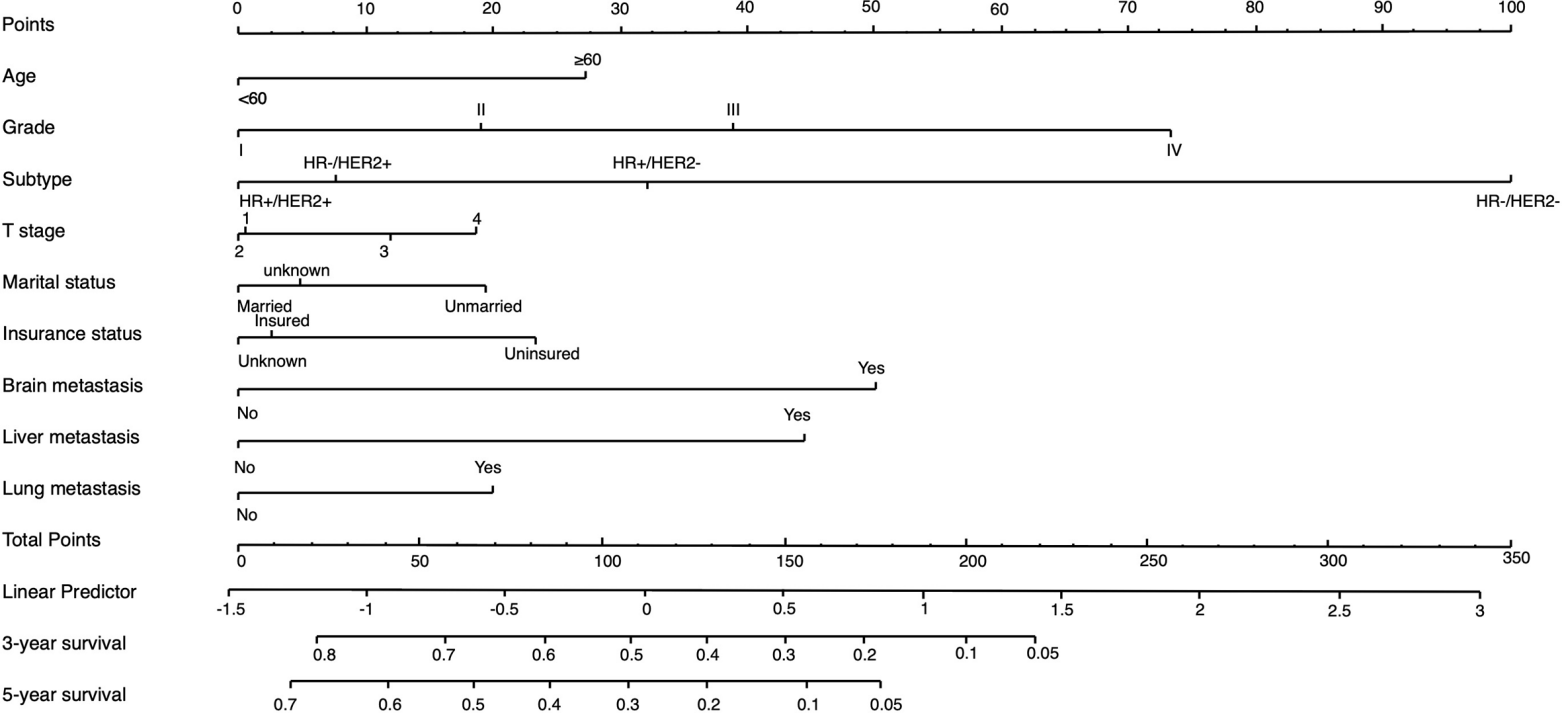
Nomogram to predict the 3-year and 5-year survival rate in metastatic breast cancer patients with initial bone involvement. Points are defined based on the prognostic contribution of the factors. Points summing the contribution of age, subtype, marital status, insurance status, brain metastasis, liver metastasis and lung metastasis are translated to the survival probability at 3 and 5 years.

**Figure 2:**
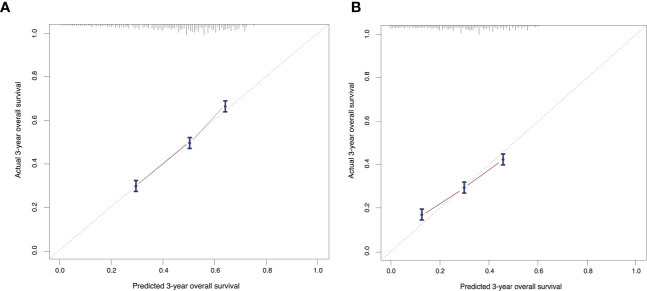
Calibration curves compare predicted and actual **(A)** 3-year and **(B)** 5-year overall survival rates. Probability of survival based on the nomogram is listed on the x-axis, while the actual probability of survival is listed on the y-axis. The calibration curves suggested that the predictive outcome have good accordance with the actual 3- and 5-year OS.

On the basis of factors independently associated with OS and BCSS, a nomogram, including age, grade, race, subtype, T stage, marital status, insurance status and visceral involvement, was developed to predict a 3-year and 5-year OS. A total nomogram score was generated for a specific patient, which was corresponded to a predicted 3- and 5-year survival (**Figure 1**). The nomogram showed medium accuracy in predicting the OS, with a C-index of 0.66 (95% CI = 0.65–0.67). The calibration curves suggested that the predictive outcome have good accordance with the actual 3- and 5-year OS (**Figure 2**).

**Figure 3:**
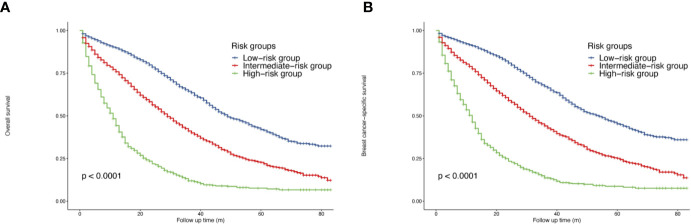
Survival of *de novo* bone metastatic patients according to different risk groups. **(A)** OS in nomogram-based low-, intermediate-and high-risk subgroups; **(B)** BCSS in nomogram-based low-, intermediate-and high-risk subgroups.

When calculated as a continuous variable, a higher nomogram score was related to a worse OS (HR = 1.01, 95% CI = 1.01–1.01, p <0.05). According to the cutoff values provided by X-tile, a risk stratification model was also generated. All the patients were divided into three groups: low-risk patients (3,092, 45.07%, total points <=86), intermediate-risk patients (2,976, 43.38%, total points 87–156), high-risk patients (792, 12.55%, total points >=157). The median OS of three groups were 49 months (95% CI = 47–53), 29 months (95% CI = 28–31) and 11 months (95% CI = 10–12), separately (p <0.05). The survival curves indicated that the risk stratification could well differentiate OS and BCSS in all subgroups (p <0.05) (**Figure 3**).

### Benefits of Primary Tumor Surgery in Patients Subdivided by Molecular Subtypes and Metastatic Sites

**Figure 4:**
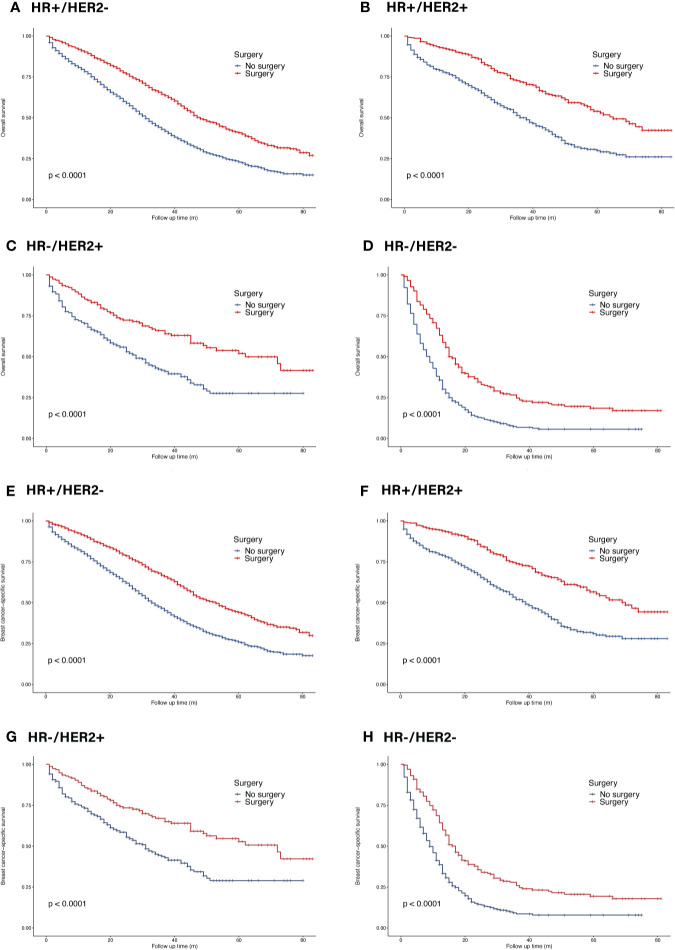
Survival of *de novo* bone metastatic patients in different subtypes according to primary surgery. **(A, E)** OS and BCSS in patients with HR^+^/HER2^−^ tumors; **(B, F)** OS and BCSS in patients with HR^+^/HER2^+^ tumors; **(C, G)** OS and BCSS in patients with HR^−^/HER2^+^ tumors; **(D, H)** OS and BCSS in patients with HR^−^/HER2^−^ tumors.

**Figure 5:**
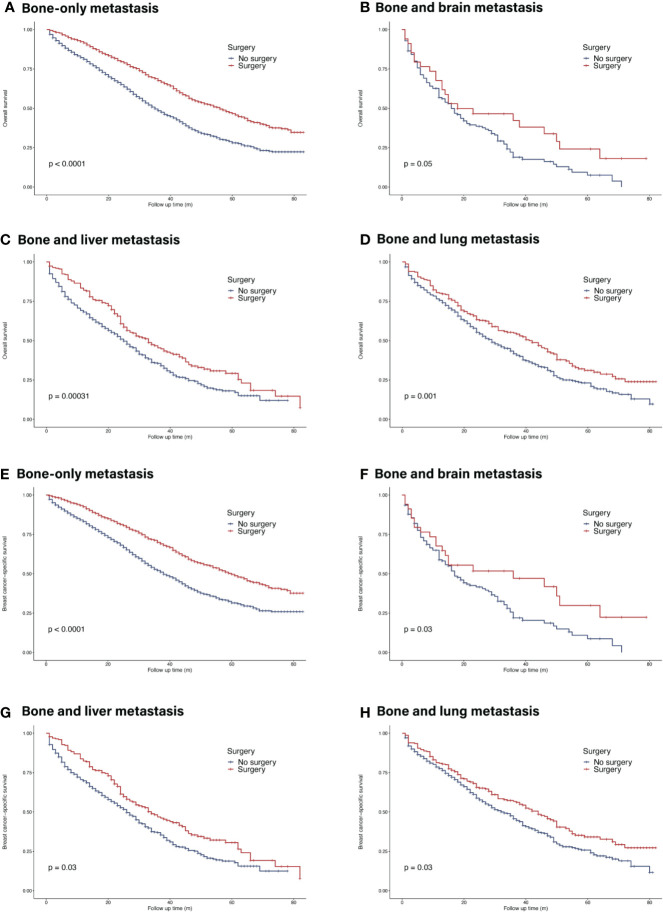
Survival of *de novo* bone metastatic patients in different metastatic burdens according to primary surgery. **(A, E)** OS and BCSS in patients with bone-only metastasis; **(B, F)** OS and BCSS in patients with bone and brain metastasis; **(C, G)** OS and BCSS in patients with bone and liver metastasis; **(D, H)** OS and BCSS in patients with bone and lung metastasis.

In the whole cohort, primary tumor surgery could prolong OS (HR = 0.56, 95% CI = 0.52–0.60, p <0.001). In terms of molecular subtypes, surgery provided extra survival benefit in all subtypes (HR^+^/HER2^−^: HR = 0.56, 95% CI = 0.51–0.61, p <0.001; HR^+^/HER2^+^: HR 0.48, 95% CI = 0.39–0.58, p <0.001 ; HR^−^/HER2^+^: HR = 0.50, 95% CI = 0.37–0.66, p <0.001; HR^−^/HER2^−^: HR = 0.50, 95% CI = 0.41–0.59, p <0.001) (**Figure 4**). In terms of metastatic burden, BOM, bone and liver metastasis as well as bone and lung metastasis patients could benefit from surgery (BOM: HR = 0.57, 95% CI = 0.52–0.63, p <0.001; bone and liver metastasis: HR = 0.70, 95% CI = 0.58–0.84, p <0.001 ; bone and lung metastasis: HR = 0.73, 95% CI = 0.60–0.88, p = 0.001). However, surgery did not significantly benefit patients with bone and brain metastasis (HR = 0.64, 95% CI = 0.40–1.02, p = 0.063) (**Figure 5**). Similarly, the analysis of BCSS showed consistent results.

### Benefits of Primary Tumor Surgery in Patients Subdivided by Nomogram Risk Category

**Figure 6:**
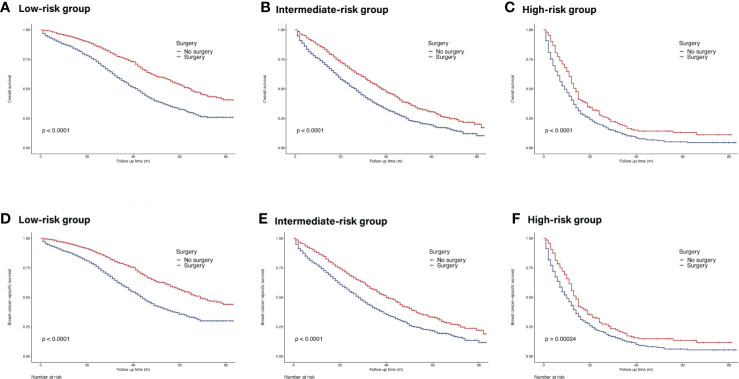
Survival of *de novo* bone metastatic patients in different nomogram-based risk groups according to primary surgery. **(A, D)** OS and BCSS in nomogram-based low-risk group; **(B, E)** OS and BCSS in nomogram-based intermediate-risk group; **(C, F)** OS and BCSS in nomogram-based high-risk group.

The Kaplan–Meier curves showed that surgery of the primary site could prolong OS in all risk subgroups (low-risk group: HR = 0.53, 95% CI = 0.47–0.59, p <0.05; intermediate-risk group: HR = 0.66, 95% CI = 0.59–0.73, p <0.05; high-risk group: HR = 0.69, 95% CI = 0.59–0.82, p <0.05) (**Figure 6**). Similar trends were achieved in BCSS.

## Discussion

With huge diversity and heterogeneity, the prognosis and treatment tactics of *de novo* stage IV breast cancer should be tailored in the light of their clinicopathological features, metastatic burden and even social status. The current study reported the prognosis of this group of patients with bone metastases according to different molecular subtypes as well as potential benefits of surgery of the primary tumor. To our knowledge, this analysis is the first population-based, retrospective, prognostic and predictive survival analysis and the first one to explore the surgical benefits of this group of patients based on subtypes and metastatic burdens. The prognostic nomogram we generalized included all the independent risk factors and show a good accuracy and accordance in predicting the survival rate of each case. The risk stratification model further differentiated patients of distinct risk subgroups, which provides critical information for indicating outcomes and facilitates individualized treatment choices.

In this analysis, several features associated with improved outcome were identified, including HR-/HER2^+^ subtype, age <60 years old, white race, lower grade, lower T stage (T ≤ T2), no concurrent visceral metastasis, married and insured status. Patients of HR+/HER2- subtypes usually present preferred prognosis among all subtypes but in our analysis, patients of HR^+^/HER2^+^ subtype (1,192/6,860) demonstrated the best outcome among all subtypes in our analysis. Similar results were reported in previous studies involving patients with various sites of metastasis. In a multicenter study held in Netherlands, the HR^+^/HER2^+^ subtype was associated with the longest survival after diagnosis of distant metastasis (HR^+^/HER2^+^ vs HR^+^/HER2^−^: HR = 0.64, 95% CI = 0.45–0.92, p = 0.02) (12). In another SEER-based analysis, HR^+^/HER2^+^ tumor was reported to have the best prognosis (HR^+^/HER2^+^ vs HR^+^/HER2^−^: HR=0.85, 95% CI = 0.77–0.94, p <0.05) (13). We postulated that several reasons may contribute to the favorable survival of HR^+^/HER2^+^ subtype. First of all, different subtypes demonstrated a totally distinguished metastatic pattern. In another SEER-based study, HR^+^ (both HER2^−^ and HER2^+^) was significantly associated with an elevated bone metastasis and better prognosis (11). Intrinsic biological characteristics and metastatic propensity of HR-positive subtype mainly contributes the good prognosis. In our subgroup analysis, we found that in different metastatic burdens, most patients with HR-positive tumors have better prognosis than those with HR-negative tumors, except patients with bone and brain metastasis (HR = 0.79, 95%CI = 0.51–1.24, p = 0.31). Secondly, the development of HER2 targeted therapy has evolved greatly. Trastuzumab, a humanized monoclonal antibody targeting HER2, reduced 44% of death risk in women with HER2+ disease compared with that of HER2- disease who did not received HER2-targeted therapy in the metastatic setting (14). In CLEOPATRA trial, the addition of pertuzumab to trastuzumab and docetaxel further improved OS in patients with HER2^+^ MBC (HR = 0.66, 95% CI 0.52–0.84, p = 0.0008) for first-line treatment (15). In progressed patients, trastuzumab emtansine could improve OS compared with capecitabine and lapatinib for second-line treatment as reported in EMILIA trial (HR = 0.75, 95% CI 0.64–0.88) (16). Thirdly, HR-positive breast cancers might display more indolent biological features than HR-negative tumors (17), and options for endocrine therapy have expanded in the last two decades. For postmenopausal patients, aromatase inhibitors (AIs) are recommended first-line endocrine therapy with or without cyclin dependent kinases 4/6 inhibitors. Multi-line endocrine modalities were available after progression or endocrine resistance in metastatic HR-positive breast cancer (18–22). Fourthly, in preclinical researches, the inhibition of HER2 could also improve endocrine sensitivity by crosstalk between HER2 and HR (23, 24). In clinical trials, the PERTAIN and ALTERNATIVE trial showed that the combination of HR and HER2 targeting therapy offers an effective and safe regimen (25, 26).

Surgery of the primary site of *de novo* MBC is a controversial topic with conflicting evidences. Many retrospective analyses of large cohort such as SEER and national cancer database (NCDB) or monocentric database have proven a better outcome of primary surgery in selected patients (5, 27–36). However, retrospective results are usually undermined for selection bias (37). Several prospective trials have also addressed this issue. A multicenter Turkish trial MF07-01 showed a statistically significant improvement in surgery arm in 5-year follow-up, especially in patients with ER/PR (+) or HER2(−) tumor, solitary bone metastasis or younger age (<55 years old) (38). An Indian randomized controlled trial in patients responsive to first-line treatment also showed that surgery could not improve OS (39). However, these prospective trials were also questioned for insufficient chemotherapy, deviation from contemporary practice, insufficient adapted p value and so on (27, 40, 41). In spite of these contradictory results, the present study suggested that in well-selected patients, primary surgery might be considered one of the treatment options.

Metastatic burden is another critical factor when making surgical decisions. The current study indicated that apart from patients with bone and brain metastasis, patients of other metastatic patterns might benefit from surgery. Likewise in a subdivision analysis of M1 patients, preferred prognosis was seen across all subdivisions after surgery except M1c category which is defined as brain involvement or multiple visceral metastasis (42). When it comes to molecular subtype, previous studies showed less benefit of surgery in metastatic triple-negative breast cancer (TNBC) patients. However, patients with bone metastatic TNBC in our cohort exhibited improved survival after primary surgery. Previous study in well-selected and risk-stratified patients demonstrated similar results in TNBC tumors (43). In summary, MBC is no more a contradiction to primary surgery, specified risk-subdivision should be employed to better screen appropriate patients for customized therapy to bring along maximum benefit.

There are some limitations of this research though. The SEER database covers about 30% of the USA population, which offers a highly representation of a general situation but on the other hand, makes it immature to apply in Asian and Chinese population on the basis of ethnic differences. In SEER database, significant confounding prognostic factors like complications, detailed treatments, treatment sequence, treatment duration, margin status, recurrence score cannot be attained, which will greatly affect the applicability of the study in real-world cases. Even though the nomogram achieved acceptable prediction and risk stratification efficacy, it lacked external validation to further enforce the reliability. The result of our analysis should be interpreted with caution and applied in well-selected cases.

In conclusion, the current study identified potential prognostic factors in predicting survival in patients with *de novo* MBC with bone metastasis and suggested primary surgery might increase survival in selected subgroup of patients. The nomogram we constructed provided a quantitative method to predict survival of individuals and well differentiated patients of different risk subgroups.

## Data Availability Statement

Publicly available datasets were analyzed in this study. This data can be found here: Surveillance, Epidemiology, and End Results (SEER) database (https://seer.cancer.gov/).

## Author Contributions

DL and JW contributed equally to the study design, data collection, statistical analysis and manuscript writing. LZ conceptualized the study and was involved in result interpretation and manuscript writing. CL, LA, and SD helped with data collection. All authors contributed to the article and approved the submitted version.

## Conflict of Interest

The authors declare that the research was conducted in the absence of any commercial or financial relationships that could be construed as a potential conflict of interest.
